# A Bibliometric Study from 1992 to 2023 on the Relationship between Biological Clock and Diabetes

**DOI:** 10.2174/0118715303316643240724095907

**Published:** 2024-08-15

**Authors:** Dayuan Zhong, Hui Cheng, Huanjie Li, Xiangbo Kong

**Affiliations:** 1 Nanhai Hospital of Traditional Chinese Medicine, Jinan University, Foshan, 528200, China;; 2 Institute of Traditional Chinese Medicine, Jinan University, Guangzhou, 510632, China;; 3 Department of Preventive Medicine, Foshan Hospital of Traditional Chinese Medicine, Foshan, 528051, China

**Keywords:** Diabetes, biological clock, bibliometrics, review, rhythm, blood glucose

## Abstract

**Aims:**

A bibliometric study was conducted to gain deeper insights into the current state of research on diabetes and the biological clock (BC).

**Methods:**

The study involved a comprehensive search for literature related to diabetes and BC published between 1992 and 2023 in the Web of Science database.

**Results:**

Ninety-five articles were published in 65 journals, with six of these journals not included in the Journal Citation Reports as of 2022. Among the remaining 59 journals, 10 had an impact factor (IF) greater than 10, and 21 had an IF greater than 5. Twenty-nine journals belonged to Quartile 1, while 16 journals were part of Quartile 2. The articles were contributed by researchers from 22 countries, with the Netherlands and the USA being the most prolific contributors. However, the total number of citations for articles from the USA was significantly higher than that of the Netherlands. The co-occurrence analysis of title and abstract keywords primarily focused on investigating the mechanisms of BC. Regarding author keywords and keyword-plus, the co-occurrence analysis centered around diabetes and BC. International collaboration was prominent among developed countries, with the Netherlands, the USA, and France being major participants. Institution-wise cooperation primarily occurred between two research institutions in the Netherlands. In total, the 95 articles received 5,157 citations, averaging 54.28 citations per article.

**Conclusion:**

To foster advancements in this area, more attention and international cooperation are necessary. Emphasizing collaborative efforts can drive the development of novel approaches to manage diabetes and regulate blood glucose levels effectively.

## 1 INTRODUCTION

Diabetes, a systemic metabolic disease, represents one of the most pressing global health challenges [1]. Over the years, its incidence has been steadily increasing, with approximately 537 million adults currently affected worldwide [1]. By 2045, this number is projected to soar to around 700 million [2]. The disease encompasses four types: type 1 diabetes (T1D), type 2 diabetes (T2D), gestational diabetes, and other forms [3]. Among these, T2D is the most prevalent, accounting for approximately 90 to 95% of all diabetic cases [4]. Despite the availability of 12 different anti-diabetic drugs [5, 6], achieving optimal blood glucose control in China remains challenging, as only 49.2% of T2D patients meet the target glycated hemoglobin level of <7.0% [7]. This indicates that relying solely on medication is insufficient for effective blood glucose control. Therefore, additional approaches, such as dietary control, lifestyle interventions, and novel BC regulation [5, 8, 9], are required to intervene in the management of blood glucose levels in diabetic patients.

The Biological clock (BC) is a molecular oscillation system that governs the rhythmicity of cellular processes [10], thereby maintaining physiological functions and body equilibrium [11]. In humans, BC primarily manifests as circadian rhythms, recurring approximately every 24 hours and subject to synchronization by environmental factors [12]. Comprising central and peripheral clocks, BC coordinates to produce daily physiological and behavioral rhythms. The central clock is located in the Suprachiasmatic Nucleus (SCN) region of the suprachiasmatic nucleus of the hypothalamus [13, 14]. BC plays a crucial role in regulating various physiological processes, including energy consumption and glucose homeostasis [15, 16]. Abnormal expression of BC genes can lead to metabolic disorders, affecting insulin secretion, glucose homeostasis, and energy expenditure [17].

Consequently, the 2020 American Diabetes Medical Diagnosis and Treatment Standard has recommended the use of dynamic blood glucose spectrum reports to assess blood glucose management in T2D patients [18], underscoring the significance of BC regulation in T2D treatment [19]. The number of articles exploring BC's role in diabetes and its complications has been steadily increasing, reflecting the growing appreciation of its importance [20]. Nevertheless, the current state of BC and diabetes-related research remains unclear. Bibliometric Analysis (BA) is an efficient method for comprehending literature-associated data, offering insights into the present status of BC and diabetes-related research, institutional collaborations, and research trends and focal points [21]. In this study, we employed BA to systematically summarize the trajectory of BC and diabetes-related research, providing a better understanding of the field's current state and areas of interest.

## 2 DATA AND METHODS

The literature search was conducted primarily in the core database of Web of Science (WOS) with a search date of June 17, 2023. The search terms used were “biological clock” and “diabetes,” and the search scope was unrestricted. The specific formula used for the literature search is as follows:

#1 “biological clock” (all field)

#2 “diabetes” (all field)

#3 #1 AND #2

### 2.1 Statistical Analysis

After completing the retrieval process, we exported the full-record citation data of the identified documents into both bibtxt and txt formats. Subsequently, we utilized the bibliometrix package for a comprehensive statistical analysis of all the literature [22]. The national function of statistical data is used to identify the number of papers published by authors from specific countries or regions. The most cited country function is employed to determine the citation counts of research published by authors from each country or region. The most relevant affiliation function is used to ascertain the number of papers published by each research institution. The statistical results were further imported into EXCEL software for secondary analysis and mapping. Additionally, to visualize the relationships between articles, we employed both the bibliometrix package and VOSviewer_1.6.15 software [23]. The visualization encompassed various aspects, including co-occurrence analysis, co-citation analysis, and cooperative network analysis. In the knowledge graph, the size of the nodes represents the number of countries, citations, keywords, references, authors, and institutions, while the thickness of the lines indicates the strength of the connections.

## 3 RESULTS

### 3.1 Analysis of the Number of Articles Published Each Year

The search in the WOS database yielded 1,061,418 diabetes-related articles and 2,555 BC-related articles. By combining the results of both searches, we identified a total of 95 articles related to both 'diabetes' and 'BC'. The specific search results are presented in Fig. (**1A**). Visualizing the 95 articles according to their year of publication, we observed that the first literature on the intersection of 'diabetes' and 'BC' was published in 1992. However, it did not garner significant attention at that time. Notably, it was only in 1997 that relevant research in this area began to gain traction. Since then, the fields of diabetes and BC have shown gradual growth, and the detailed trend of literature publication is depicted in Fig. (**1B**).

### 3.2 Analysis of Journals

Ninety-five articles were published across 65 journals, with only 13 journals publishing more than 2 articles related to 'diabetes' and 'BC'. The journal with the highest number of publications is “diabetes” with 11 articles, followed by “chronobiology international” with 5 articles, and “international journal of molecular sciences” with 4 articles, as depicted in Fig. (**2A**). Among the 65 journals, six were not included in the 2022 Journal Citation Reports (JCR), thus lacking an Impact Factor (IF), category, and IF Quartile information. Among the remaining 59 journals, 56 were part of the Science Citation Index Expanded (SCIE), 2 were included in the Emerging Sources Citation Index (ESCI), and 1 was covered by the Social Sciences Citation Index (SSCI).

In this set of 59 journals, 10 had an IF greater than 10, while 21 had an IF between 5 and 10. Additionally, when considering quartiles, 29 journals were classified under Quartile 1, 16 journals under Quartile 2, 10 journals under Quartile 3, and 4 journals under Quartile 4, as illustrated in Fig. (**2B**). Moreover, these 59 journals represented a diverse range of 21 categories, with the largest representation in the category of “endocrinology & metabolism” with 11 journals, followed by “nutrition & dietetics” with 7 journals, and “biochemistry & molecular biology” with 6 journals, as shown in Fig. (**2C**).

### 3.3 Analysis of Countries and Institutions

The 95 articles involved contributions from 22 countries, with the Netherlands and the USA being the most prolific, each accounting for 19 articles, as illustrated in Fig. (**3A**). Regarding citations, the USA received the highest number of citations (1773), followed by the Netherlands with the second-highest number of citations (1102), as depicted in Fig. (**3B**). Interestingly, when considering the average number of citations per article, Brazil had the highest average (153), with the USA coming in second (93), as shown in Fig. (**3C**).

In terms of institutions, the University of Amsterdam took the lead with the largest number of publications (26articles), closely followed by the Netherlands Institute for Neuroscience, which contributed the second-largest number of articles (17 articles), as displayed in Fig. (**3D**).

### 3.4 Co-occurrence Network Analysis

The analysis of co-occurrence words in the titles of the 95 articles revealed a diverse range of topics, indicating a broad scope of research content. Certain words appeared more frequently together, such as “clock,” “circadian,” “dopamine activity,” “cellular redox state,” “management,” and “ghrelin,” as depicted in Fig. (**4A**). In the abstracts of the articles, the co-occurrence word analysis highlighted the prevalence of certain words like “clock,” “mechanism,” “gene,” “cell,” “impact,” “risk,” and “light,” indicating their frequent association, as shown in Fig. (**4B**). Furthermore, the results of the keyword co-occurrence analysis demonstrated that “biological clock” often co-occurs with “circadian rhythm,” “diabetes,” “circadian rhythms,” and “adipose tissue,” as displayed in Fig. (**4C**). Similarly, in the keyword-plus co-occurrence analysis, “gene-expression” was found to frequently co-occur with “food-intake,” “metabolic syndrome,” “circadian clock,” and “high-fat diet,” as illustrated in Fig. (**4D**).

### 3.5 Cooperative Network Analysis

The findings from the country's cooperation network analysis indicate relatively limited collaboration between different countries. Notably, the USA frequently collaborates with Spain and Canada. Meanwhile, the Netherlands shows a tendency to partner with France and the People's Republic of China, as displayed in Fig. (**5A**). Institution-wise network analysis reveals notable collaborations between specific institutions. For instance, the Netherlands Institute of Neuroscience engages in regular partnerships with the University of Amsterdam, the Netherlands Institute for Brain Research, and the National Autonomous University of Mexico. Brigham & Women's Hospital shows collaborations with Harvard University, the University of Lille, the University of Murcia, and Tufts University, as demonstrated in Fig. (**5B**).

## 4 DISCUSSION

The prevalence of diabetes in China continues to rise due to changes in the lifestyles and dietary habits of the residents. A cross-sectional study of adults aged 18 years and older in mainland China revealed that the estimated prevalence of diabetes increased from 10.9% in 2013 to 12.4% in 2018 [24]. Projections indicate that by 2030, the estimated prevalence of diabetes in China will reach 19.8% [25]. Additionally, the combined prevalence of diabetes and prediabetes is at 50.5% [24]. Currently, China has the largest number of diabetic patients in the world, accounting for more than a quarter of global cases [1]. This surge in the diabetic population poses a significant burden on the healthcare system [25].

Type 2 diabetes is characterized by persistent hyperglycemia and absolute or relative insulin deficiency [26]. Insulin secretion is regulated by the circadian system, with higher secretion during the day and relative reduction at night [27]. This highlights the role of the biological clock (BC) in blood glucose and insulin homeostasis regulation. Irregular lifestyles, such as improper sleep and shift work, can disrupt the BC, leading to conditions like type 2 diabetes [28, 29]. As research advances, it has become evident that mammalian BC consists of central BC and peripheral BC [30]. The central BC is located in the suprachiasmatic nucleus (SCN) of the hypothalamus and is responsible for generating and maintaining the body's daily cycle rhythm. The information it sends out controls various physiological rhythms, including sleep, body temperature, and endocrine functions [30]. Peripheral BC exists in tissues like muscles, liver, and pancreas. While it cannot independently generate physiological rhythms, it is regulated by other signaling molecules under the control of SCN [31-33]. This regulatory mechanism governs the rhythmic patterns of blood glucose and insulin in the body. Proper management of this relationship can aid in preventing obesity and other metabolic syndromes [34-36]. As research progresses, the understanding of the relationship between diabetes and BC has grown.

In the bibliometric study, a total of 95 articles were retrieved. The literature on BC and diabetes was first published by Rubin in 1992 [37]. The study by Rubin *et al*. focused on DNA synthesis and mitosis in the intestinal epithelium, demonstrating circadian rhythms even in fasted rats [37]. Over time, this article has been cited 192 times, with an average of 6.19 citations per year. The annual trend indicates a gradual increase in publications related to diabetes and BC, but the overall number of publications per year remains relatively low, seldom exceeding 10. This suggests ample opportunities for further research in the field of diabetes and BC.

A total of 95 articles were published in 65 journals, with the highest number of articles appearing in “Diabetes.” This journal, as the official publication of the American Diabetes Association, holds significant influence in the field. According to the 2022 Journal Citation Reports (JCR), “Diabetes” boasts an impressive Impact Factor (IF) of 7.70, ranking 18th out of 145 diabetes journals, and is classified under JCR's Quartile 1. Moreover, it also falls within the top tier of diabetes journals in the Chinese Academy of Sciences (CAS) Journal Ranking, reaffirming its importance in the study of diabetes and BC. Among the 65 journals, 59 are indexed in the 2022 JCR, with 29 belonging to Quartile 1 and 16 to Quartile 2. These Quartile 1 and 2 journals enjoy higher international recognition, emphasizing the significance of articles related to diabetes and BC.

The 95 articles involved authors from 22 countries, with the Netherlands and the USA contributing the most publications. However, the USA's articles garnered substantially more citations than those from the Netherlands, highlighting their greater influence and significant contributions to the field of diabetes and BC. Notably, the Universiteit van Amsterdam and the Netherlands Institute for Neuroscience emerged as the institutions with the highest number of publications, indicating their deep involvement in research on diabetes and BC. Co-occurrence word analysis indicated that the title and abstract words primarily focused on exploring the mechanisms of BC. Similarly, the keyword and keyword-plus co-occurrence analysis revealed that the retrieved literature was closely related to diabetes and BC, reaffirming the relevance of the study's topic. As the number of published articles was not extensive, international cooperation was mainly centered around developed countries, such as the Netherlands, the USA, and France. The collaboration between institutions predominantly took place within two research institutions in the Netherlands, indicating a limited amount of international cooperation.

In total, the 95 articles received 5,157 citations, with an average of 54.28 citations per article. The most frequently cited article was a research paper by Liu C published in Neuron in 1997 [38]. Liu *et al*. discovered that BC is most sensitive to light phase shift activity during the circadian rhythm, with melatonin playing a role in inhibiting night phase shift. Surprisingly, the study found that the Mel1a melatonin receptor is not essential for the phase shift of melatonin in mice. The second most cited article was by Horwitz published in Nature Genetics in 1999 [39]. Horwitz *et al*. found that the interaction between neutrophil elastase and Selpinsh or other substrates was disrupted, potentially regulating the mechanism of controlling hematopoietic BC time. The third most cited article was published by Narasimamurthy in Proceedings of the National Academy of Sciences of the United States of America in 2012 [40]. Narasimamurthy *et al*. discovered that the absence of the core clock component protein CRY protein might relieve its inhibition of cAMP production, leading to increased cAMP and PKA activation, which, in turn, triggers NF-κB activation by phosphorylating p65 at S276. This mechanism may be the link between circadian rhythm disorders and increased susceptibility to chronic inflammatory diseases, as shown in Table **1**.

## 5 LIMITATIONS

There are several limitations to acknowledge in this study. Firstly, we solely relied on the WOS database for literature inclusion, potentially overlooking relevant articles published in other databases. Consequently, some important contributions to the research topic might have been missed. Secondly, despite our rigorous search and screening process, a few unrelated articles might have been inadvertently included, suggesting the possibility of some omissions. Thirdly, our analysis of articles related to diabetes and BC was brief and did not encompass the latest advancements, depth, and existing research challenges in this field. As a result, our study's capacity to provide comprehensive guidance for the future development of this domain is limited. Further comprehensive research is needed to address these limitations and enhance the understanding and progress in the field of diabetes and BC.

## CONCLUSION

Despite the limitations of this study, we conducted a relatively comprehensive search of diabetes and BC-related literature. We systematically organized and examined the published articles pertaining to diabetes and BC, exploring their key characteristics in terms of publication year, journals, countries, institutions, titles, abstracts, keywords, and highly cited articles. As a result, this study provides valuable insights and guidance for future research in the field of diabetes and BC. It lays the foundation for further exploration and advancement in this important area of study.

## AUTHORS’ CONTRIBUTIONS

H.L., X.K., H.C. contributed to the study conception and design of the manuscript and D.Z. helped writing the paper.

## Figures and Tables

**Fig. (1):**
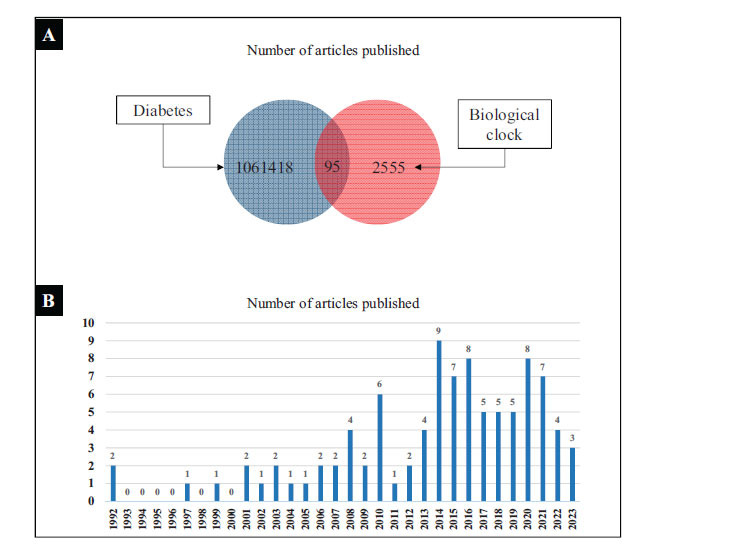
(**A**). Results of literature search. (**B**). Number of publications per year.

**Fig. (2):**
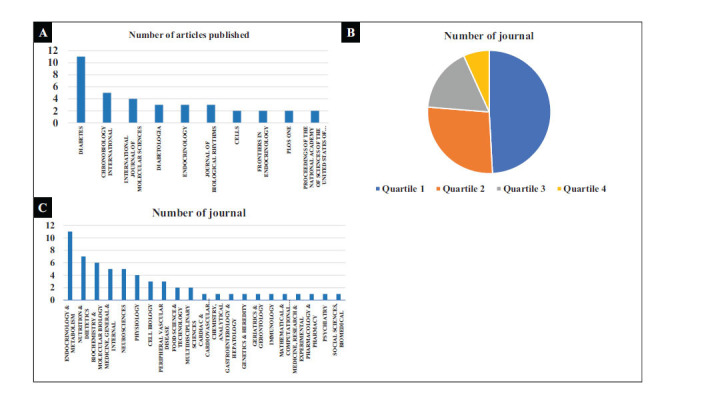
(**A**). Journals with more than 2 articles published. (**B**). JIF Quartile distribution of journals. (**C**). Category of journals.

**Fig. (3):**
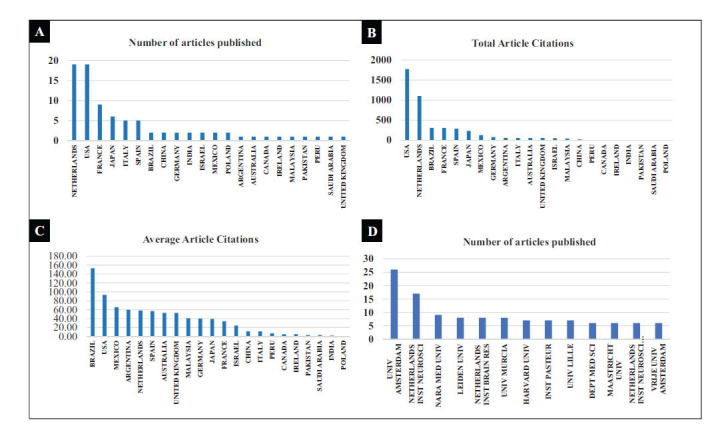
(**A**). 22 The number of papers published in 22 countries. (**B**). 22 The number of citations of papers published in 22 countries. (**C**). 22 The average number of citations of papers published in 22 countries. (**D**). The number of papers published > 5 scientific research institutions.

**Fig. (4):**
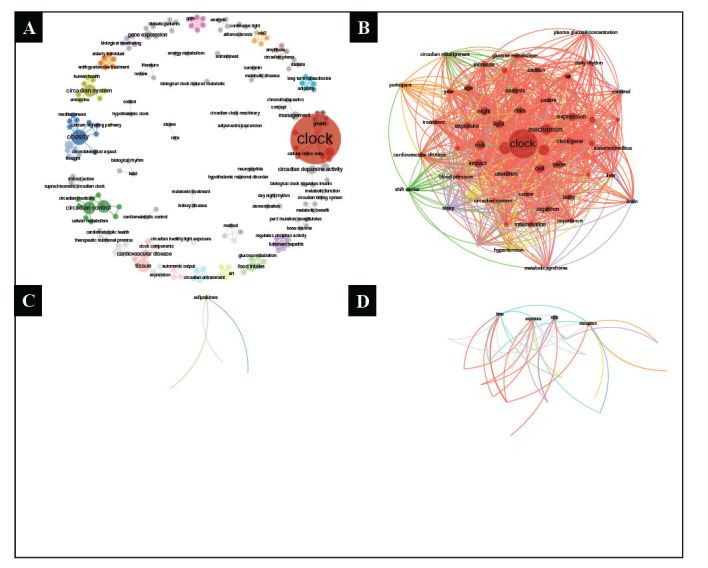
(**A**). Title co-occurrence word analysis results. (**B**). Abstract co-occurrence word analysis results. (**C**). Author keyword co-occurrence analysis results. (**D**). Keyword-plus co-occurrence word analysis results.

**Fig. (5):**
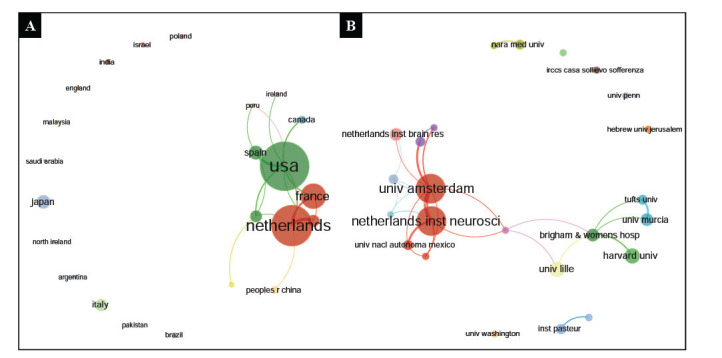
(**A**). Country cooperation network; (**B**). Institution cooperation network.

**Table 1:** The 10 most frequently cited articles.

**Author**	**Year**	**Journal**	**DOI**	**Total Citations**
LIU C	1997	NEURON	10.1016/S0896-6273(00)80350-5	564
HORWITZ M	1999	NATURE GENET	10.1038/70544	321
NARASIMAMURTHY R	2012	PROC NATL ACAD SCI U S A	10.1073/pnas.1209965109	275
LA FLEUR SE	2001	DIABETES	10.2337/diabetes.50.6.1237	241
BROWN EN	1992	J BIOL RHYTHMS	10.1177/074873049200700301	192
WRIGHT KP	2001	PROC NATL ACAD SCI U S A	10.1073/pnas.201530198	173
KREIER F	2006	ENDOCRINOLOGY	10.1210/en.2005-0667	128
CHAPPELL PE	2003	J NEUROSCI	10.1523/JNEUROSCI.23-35-11202.2003	126
RUITER M	2003	DIABETES	10.2337/diabetes.52.7.1709	125
SIMONS RL	2016	SOC SCI MED	10.1016/j.socscimed.2015.12.001	120

## LIST OF ABBREVIATIONS

BC: Biological Clock
CAS: Chinese Academy of Sciences
ESCI: Emerging Sources Citation Index
IF: Impact Factor
JCR: Journal Citation Reports
SCIE: Science Citation Index Expanded
SSCI: Social Sciences Citation Index
T1D: Type 1 Diabetes
T2D: Type 2 Diabetes
